# An Utstein style based on a reporting system of the Emergency Department’s cardiopulmonary resuscitation in an Emergency Hospital in Romania

**Published:** 2013-12-25

**Authors:** O Tudorache, S Armean, V Georgescu

**Affiliations:** * ”Sf. Pantelimon” Emergency Hospital; ** ”Iuliu Hatieganu” University of Medicine and Pharmacy; *** ”Carol Davila” Nephrology Hospital

**Keywords:** cardiac arrest, Utstein style, resuscitation

## Abstract

Abstract

Rationale: Different Utstein based reporting systems are used for research purpose in resuscitative medicine worldwide and resuscitation attempts data are available from different countries. In Romania, the only data available has been from the previous work in the Emergency Department of “Sf. Pantelimon” Emergency Hospital, since 2006.

Objective: This study was conducted to describe the characteristics of the cardiac arrest event in our Emergency Department (ED) and to identify factors associated with the outcome and the event itself.

Methods and Results: This descriptive study refers to the resuscitation attempts performed in the ED of our hospital between January 1st 2011 and December 31st 2011, following the Guidelines of the European Resuscitation Council 2010. The data used were gathered from the observational sheets. The reporting form used is Utstein-based, referring to the patient characteristics, event and outcome. A number of 81 cases have been analyzed; in 33 cases (40.74%), the cardiac arrest occurred out of the hospital. The return of the spontaneous circulation (ROSC) occurred in 25 patients (30.86%), with 12% discharged alive. The most common cause of arrest was the myocardial infarction. The first rhythm monitored was non-shockable in 92.59% of the cases. However, 24 patients developed a shockable rhythm at some point during the resuscitation and 37.5% of these experienced ROSC.

Discussion: The Utstein- based reporting system used provides a standardized, comprehensive method for data collection. Further research is needed in order to obtain valuable data with statistic relevance. Conclusions related to the aspects of the population in the area the hospital serves can be drawn.

## Introduction

The cardiac arrest represents a dramatic event that ends the life of every human being. Sometimes it is expected, following a chronic terminal illness, sometimes it is totally unexpected, due to trauma, unknown medical issues or an acute turn of a medical condition under treatment. To restore life in a person in clinic death is a major day-to-day objective in every Emergency Department.

However, despite all the gestures made to restart the heart and to obtain the best cerebral performance in the situation given, resuscitated patients may suffer from severe ischemic brain injury with a poor neurological outcome [**1**].

Over the past years, studies have been published reporting diverse outcomes in resuscitation attempts for the hospital cardiac arrest (IHCA) and out of the hospital cardiac arrest (OHCA). In 2003, “The National Registry on Cardiopulmonary Resuscitation”, the largest registry on cardiac arrest at that time, reported the Return of Spontaneous Circulation (ROCS) in 44% of the resuscitation attempts related to the in-hospital cardiac arrests. In 17% of these, survival to discharge has been noted [**2**]. These results have been obtained in hospitals that had very well implemented resuscitation protocols.

Data about resuscitation attempts, both from IHCA and OHCA, are available from different countries. Few studies were conducted in Romania and the only data available is from the previous work in the Emergency Department of ”Sf. Pantelimon” Emergency Hospital from 2006: ROSC was achieved in 63.8% and 14.1% of the patients were discharged alive [**3**]. In 2010, the Romanian Registry on Cardiac Arrest (RRCA) has been established, promising to gather valuable information about IHCA and OHCA in Romania [**4**,**5**]. The cases selected for this study are also going to integrate in the RRCA for a future wider view regarding resuscitation in our country.

## Materials and methods

This paper refers to a descriptive study of the resuscitation attempts performed in the Emergency Department of ”Sf. Pantelimon” Emergency Hospital between the 1st of January 2011 and 31st of December 2011.

The hospital involved in the study, ”St Pantelimon” Emergency Hospital, is a major, teaching Emergency Hospital in Bucharest, for adult population, affiliated to “Carol Davila” University of Medicine and Pharmacy. The Emergency Department counted a number of 82.288 presentations in the period indicated. The personnel of the Emergency Department consists of physicians trained in Emergency Medicine, consultants, specialists and resident doctors, all trained in Advanced Life Support (ALS) by the Romanian Resuscitation Council, part of them being instructors in ALS for the European Resuscitation Council (ERC). The Romanian Resuscitation Council’s instructors also train the nurses working in the department, and all the resuscitation procedures in the Emergency Department have been conducted according to the 2010 ERC Guidelines.

There were teams of different levels from the Emergency Medical System (EMS) to reach the case for the Out of Hospital Cardiac Arrests (OHCA). Regardless of the EMS level (paramedics only, with nurse, with doctor), all OHCA received chest compression and ventilation with supraglottic devices. The teams led by doctors completed the ALS protocol (with drugs and orotracheal intubation) on the scene of the cardiac arrest.

A medical doctor involved in the research, using data from the observational sheets, having institutional approval and respecting all the regulations related, gathered the information. The reporting form used is Utstein-based, having as main sections, data referring to patient characteristics (demographic, known co-morbidity), event (including resuscitation algorithm) and outcome.

**Inclusion criteria**

All the patients who received CPR in the Emergency Department, performed by the ED personnel, were included. Patients who were already admitted on any ward of the hospital, but received CPR from the ED personnel (as in our hospital, the role of the resuscitation team is assumed by the emergency physician on call), have been excluded from the study.

The OHCA were included in the study only if the patients were presented by the EMS personnel with ongoing CPR and the cases were solved in the ED, or, if patients resuscitated in pre-hospital suffered another cardiac arrest in our ED.

## Results

**Patient data**

In 2011, a number of 81 patients with cardiac arrest met the inclusion criteria in the study. We have excluded the patients already deceased when being presented and the DNAR cases.

We included a total number of 81 patients, aged 0-91, median age being 70 years. ”Sf. Pantelimon” Emergency Hospital is serving adult patients, so we only counted a number of 2 children in our study (2.46%).

In 33 cases (40.74%), the cardiac arrest occurred out of hospital, and the patients were brought by the ambulance with ongoing CPR, the case being solved by the ED personnel; none of these patients survived to discharge.

A number of co-morbid diseases have been noticed; the most common of them and other significant patient related data are summarized in **Table 1**.

**Table 1:**
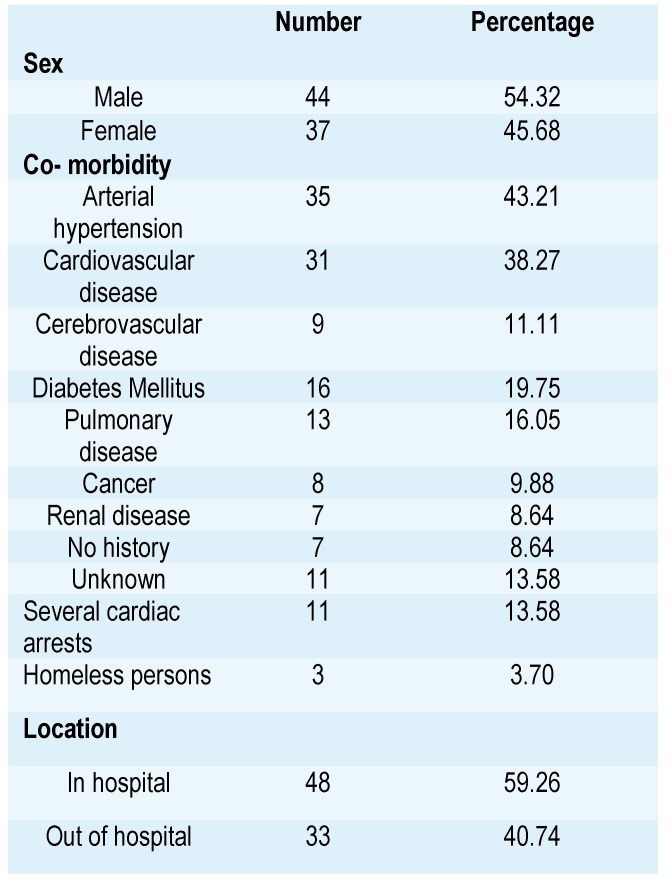
Patient related data

**Event data**

All the cardiac arrests that occurred in the ED (59.25%) have been whiteness and action has been taken immediately. Oro-tracheal intubation has been used for airway management as first choice, appropriate drugs have been given and quality chest compressions have been performed.

In a number of 33 cases, the cardiac arrest occurred out of hospital (OHCA), and we learned from the report charts that 31 cases had been witnessed, and bystander CPR has been performed only in 17 cases. However, in all cases of OHCA in which CPR has been performed, the EMS personnel, paramedics, nurses or doctors had performed it. No layperson CPR has been noted. None of the OHCA survived to discharge.

**Fig. 1:**
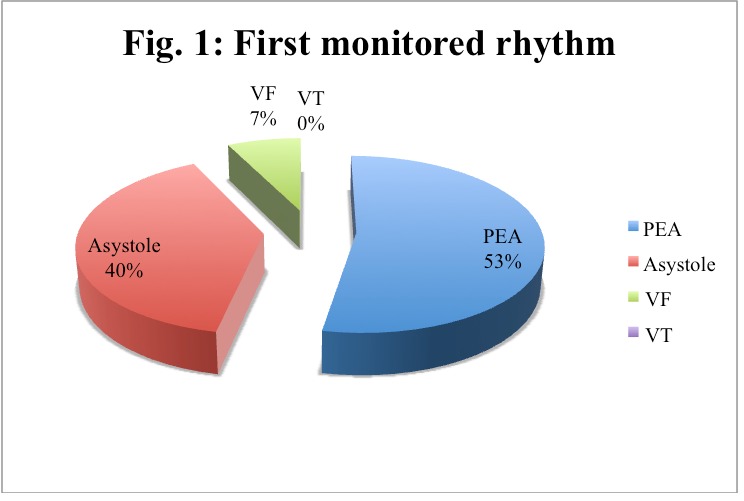
First monitored rhythm

About the first monitored rhythm, we found that 92.59% were non-shockable rhythms (**Fig. 1**). However, 24 patients developed a shockable rhythm at some point during the resuscitation and 37.5%% of these (9 patients) had pulse after the electric shock.

Regarding the causes of cardiac arrest, we found the myocardial infarction to be the most common, being identified in 29 patients; 19 patients presented respiratory failure, 4 patients experienced a traumatic event and 15 cases remained with an unknown cause for cardiac arrest (**Fig. 1**). Details about the underlying cause of cardiac arrest for the study group are shown in **Table 1**.

**Fig. 2:**
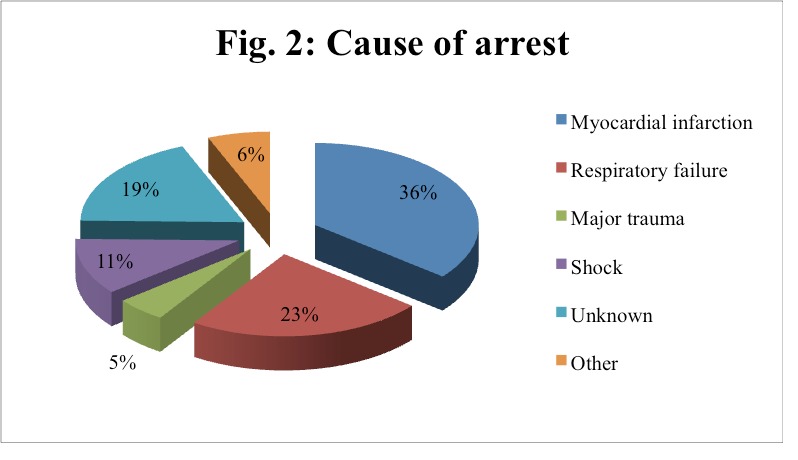
Cause of arrest

**Table 2:**
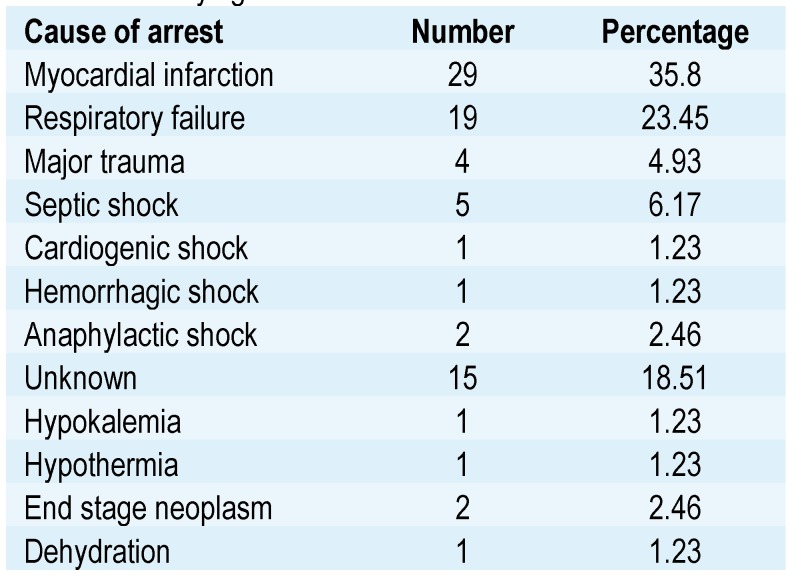
Underlying cause of arrest

**The resuscitation algorithm**

Regarding the airway management, all patients underwent orotracheal intubation. A number of 23 patients had the airway secured before the cardiac arrest, as their condition was already poor at presentation in the Emergency Department, and the doctors anticipated the tragic event. None of the patients received mouth-to-mouth ventilations, and the supraglottic devices were used in the OHCA managed by EMS without a doctor in the team, only until the presentation in the ED.

24 patients presented a shockable rhythm at some point during the resuscitation and defibrillation attempts were provided to all of them. 9 of the patients had pulse after a defibrillation (37.5%). However, 8 of the patients receiving electrical shocks also received amiodarone during resuscitation, as they presented with refractory ventricular fibrillation. Only one of these patients had pulse at some point.

All patients received intra-resuscitation drugs, adrenalin being the one common in all resuscitation attempts. For prolonged resuscitations, bicarbonate has been given; dopamine, dobutamine and atropine have also been mentioned in the charts. Amiodarone has been given in all ventricular fibrillations refractory to the electric shock, but also, in some patients, before the cardiac arrest, to treat arrhythmias.

About the resuscitation time, for all 56 irresuscitable cases, the resuscitation attempts ended if 30 minutes of ongoing Asystole was noted during the full- protocol resuscitation. For the resuscitated cases (25 cases), we noted the minutes of CPR until ROSC, and it varies from 3 minutes to 42.

**Outcome**

**Fig. 3:**
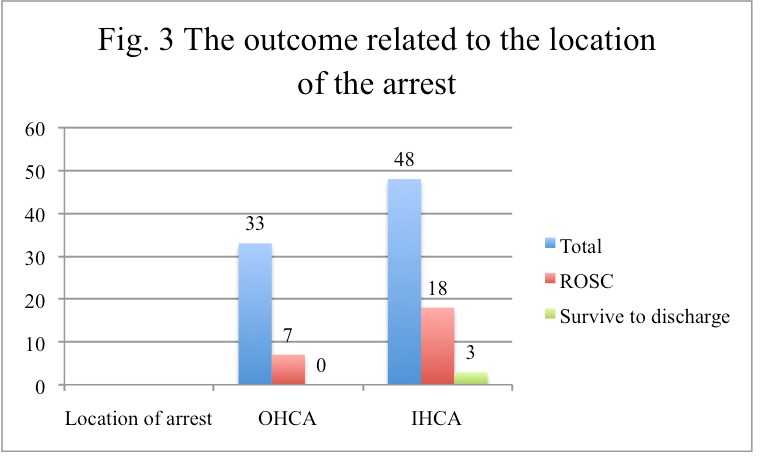
The outcome related to the location of the arrest

We noted a 30.86% success in the resuscitation attempts; out of the 81 cases analyzed, 25 patients experienced ROSC, after being resuscitated in the ED. However, only 3 patients were discharged alive (12%), but all of them with the best Cerebral Performance Category (CPC of 1). There is also evidence that these 3 patients survived for at least 3 months, as routine medical checks have been found for these patients later on (**Fig. 3**). None of these patients received any electrical shock during resuscitation.

About the resuscitated patients, 22 of them died in the Intensive Care Unit during the post-resuscitation care. We can observe from the data in Table 3 the relation between the minutes of CPR and the distance survival. 

**Table 3:**
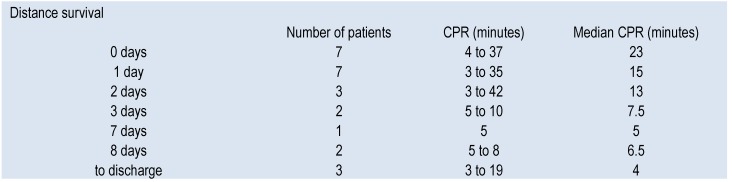
Distance survivals

## Discussion

In this study, we described the process and the outcome of the resuscitation attempts over the year 2011, in major Emergency Department in a teaching Clinical Emergency Hospital in Bucharest, Romania. 

The Utstein-based reporting system used provides a standardized, comprehensive method for data collection. Utstein-based templates have extensively been used in studies regarding the cardiac arrest [**6**]. The system has its limitations, as difficulties in filling the data have been reported, and a large, yet to be explained variability in outcome has been noted [**7**]. 

Comparing the results to the other data available from the same Emergency Department, we noticed that there was a small difference between the outcomes: 12% of the patients were discharged alive and were noted in our study, compared to 14.1% communicated by Georgescu V. et al for the period 2001-2005 [**3**]. 

The primary cause of cardiac arrest in the area served by “Sf. Pantelimon” Hospital remains the myocardial infarction. 

Regarding the international data, we found that there is an important difference between the outcome of studies regarding the OHCA and those regarding the IHCA. 

In a pilot study, meant to start the Slovak registry, 6.7% one-month survival was noted for OHCA [**8**]. For the IHCA, the scores for “discharged alive” are higher, such as 18% reported by a study in Taiwan [**9**], or 17%, reported by NRCPR, a large US registry [**10**]. 

It should be noted that an important part of the IHCA events described in these studies happened in intensive care units or in coronary care units. A research about IHCA in unmonitored wards (Internal Medicine) shows a 2.4% survival rate to discharge [**11**]. 

Having Emergency Departments (ED) and Emergency Mobile Units with emergency physicians as staff, gives a particularity to the cardiac arrest event in Romania. 

If being witnessed by a medical team, the OHCA cardiac arrest may use similar drugs and human resources as the cardiac arrest that occurred in the ED. However, education for laypersons in the field of resuscitation in our country is much needed, as none of the OHCA included in the study benefited from bystander CPR. 

**Disclosure**: none
